# Detailed analysis of Ethereum network on transaction behavior, community structure and link prediction

**DOI:** 10.7717/peerj-cs.815

**Published:** 2021-12-10

**Authors:** Anwar Said, Muhammad Umar Janjua, Saeed-Ul Hassan, Zeeshan Muzammal, Tania Saleem, Tipajin Thaipisutikul, Suppawong Tuarob, Raheel Nawaz

**Affiliations:** 1 Department of Computer Science, Information Technology University, Lahore, Pakistan; 2 Department of Computing and Mathematics, The Manchester Metropolitan University, Manchester, United Kingdom; 3 Faculty of Information and Communication Technology, Mahidol University, Salaya, Nakhon Pathom, Thailand; 4 Department of Operations, Technology, Events and Hospitality Management, Manchester Metropolitan University, Manchester, United Kingdom

**Keywords:** Ethereum, Graph Neural Network, Wealth Distribution, Network Community Structure

## Abstract

Ethereum, the second-largest cryptocurrency after Bitcoin, has attracted wide attention in the last few years and accumulated significant transaction records. However, the underlying Ethereum network structure is still relatively unexplored. Also, very few attempts have been made to perform link predictability on the Ethereum transactions network. This paper presents a Detailed Analysis of the Ethereum Network on Transaction Behavior, Community Structure, and Link Prediction (DANET) framework to investigate various valuable aspects of the Ethereum network. Specifically, we explore the change in wealth distribution and accumulation on Ethereum Featured Transactional Network (EFTN) and further study its community structure. We further hunt for a suitable link predictability model on EFTN by employing state-of-the-art Variational Graph Auto-Encoders. The link prediction experimental results demonstrate the superiority of outstanding prediction accuracy on Ethereum networks. Moreover, the statistic usages of the Ethereum network are visualized and summarized through the experiments allowing us to formulate conjectures on the current use of this technology and future development.

## Introduction

Networks are ubiquitous data structures representing complex real-world scenarios that generally involve relationships among objects (Hamilton, 2020). Blockchain is one of the promising networks that have the potential to reform several conventional businesses. The first generation of blockchain, namely Bitcoin, has demonstrated that the global consensus can be completed without a trusted third party or central authority. As a result, many researchers have put a lot of effort into designing more powerful and multifunctional blockchain systems due to their high applications in numerous real-world settings.

Later, Ethereum (a system of a transaction-based state machine and a fully decentralized peer-to-peer) was developed in 2015 and became the second-largest blockchain platform, where the market value reached over 1,000 million dollars in 2020 (Nakamoto, 2019; Wood, 2014; Ma et al., 2021; Akhtar et al., 2021). After the development of Ethereum, it has been successfully used in a variety of applications, including transaction management, smart contracts, and industrial applications. Since Ethereum’s growth in value and adoption in the market, critical enterprise applications based on programming frameworks, and the total number of users is increasing, the research community’s attention is now focused on investigating and analyzing various aspects of the Ethereum system (Wu et al., 2019).

Although various statistical analyses on blockchain transactional networks have been performed, most of these methods focus on deanonymization (Androulaki et al., 2013; Ober, Katzenbeisser & Hamacher, 2013; Said et al., 2019), clustering (Meiklejohn et al., 2013; Said et al., 2018), and finding malicious activities (Hirshman, Huang & Macke, 2013; Harlev et al., 2018; Möser, Böhme & Breuker, 2013; Ao et al., 2021; Rodriguez-Garcia, Sicilia & Dodero, 2021) of Bitcoin system. However, such Bitcoin data analysis cannot be applied or performed directly on the Ethereum data because of the different protocols and designs.

**Figure 1:**
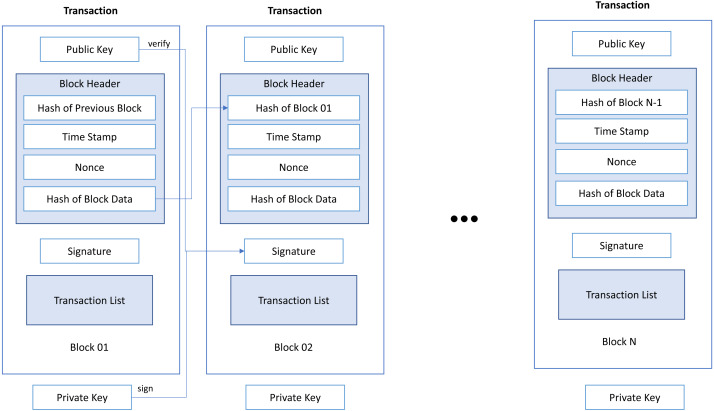
The structure and transaction of Ethereum blockchain.

Ethereum users’ activities are encapsulated in the blocks as shown in Fig. 1 where each transaction inside a block includes the sending and receiving addresses and the transferred value. As an open shared ledger, Ethereum allows any user to store the history of the entire transaction. By using this history, special nodes (miner’s node) can confirm new transactions. Miner’s integrity is determined by a proof mechanism that validates miners’ transactions. It notifies new transactions added to the Ethereum chain *via* blocks added at a constant rate between 10 and 20 s (Gervais et al., 2016).

Ethereum is difficult to calculate when changing a transaction (double spending) (Rosenfeld, 2014) that a user has already used since the processing information for all relevant blocks must be re-executed. All users of the Ethereum network receive and send transactions through ID or address generated by the Elliptic Curve Digital Signature Algorithm (ECDSA), which gives the private and public key pairs. The private key is used to send transactions to another address, and the public key is used to receive transactions from another address. Ethereum users can synchronize the nodes with the network to get information about every transaction. A transaction includes sender address, recipient address, amount (Ether), time, and other attributes as shown in Table 1. However, for security and anonymity, a user’s real identity is not tied to an address, making analysis difficult.

**Table 1:** Block and transactions’ attributes of the Ethereum data.

Attribute	Description
Block Information	
name	A unique block identifier
nonce	A hash of proof-of-work
hash	A unique hash of the block
miner	A beneficiary address who receives mining reward
total Difficulty	Indicating the total difficulty of the chain up to a specified block by an integer value
difficulty	Specifying the difficulty level by an integer value
extraData	A field containing additional data from a block
size	The block size in bytes
gasUsed	Total gas used by all transactions in a block
gasLimit	Maximum gas usage of all transactions in a block
timestamp	A UNIX timestamp when blocks were contrasted
transactions	Unique ID of the transaction or a hash array of 32-byte transactions
uncles	Uncle block hashes array

Transactional Information	
nonce	Before that transaction, total transactions made by similar sender
hash	A unique transaction hash
blockNumber	A unique block number for the committed transaction block
blockHash	A unique hash for the committed transaction block
from	A unique hash string considered as sender’s address
to	A unique hash string considered as receiver’s address, resulted null if creating contract is the purpose of received transaction
value	The transferred amount in (Wei) where Wei is unit of Ethereum
gasPrice	Sender provided gas proice in (Wei)
gas	Sender provided gas amount
input	Extra data sent with the transaction

Existing studies on Ethereum focus on the analysis of the transactional Ethereum data in terms of quantity, network in-degree, and out-degree distributions. For example, Muzammal et al. (2019) deployed the Decision Tree algorithm to predict future transactions by utilizing two features: “from” and “to”, which demonstrated the capability of using the network theory to analyze the Ethereum transactional network. However, most studies in this area still overlooked detailed analyses of the network community structures. While extensive studies have been performed on blockchain networks such as Bitcoin (Nerurkar et al., 2021) due to its long establishment, network analyses on Ethereum are quite limited (Li et al., 2020). Such analyses could play a crucial role in wealth distribution, the network’s relational structure, and the link predictability from heterogeneous network data.

This paper presents a sequence of studies on the Ethereum network, including detecting community structures and investigating link predictability on the transaction network using a graph structure learning technique. Specifically, we propose a **D**etailed **A**nalysis of Ethereum **Net**work on Transaction Behavior, Community Structure, and Link Prediction framework, namely DANET, as a unified platform to conduct various analyses simultaneously. Specifically, DANET consists of four main modules: (1) Ethereum Data Management; (2) Ethereum Transaction Behavior Analysis; (3) Ethereum Community Structure Analysis; and (4) Ethereum Link Prediction Analysis. In particular, Ethereum Data Management is designed to collect and filter the transactional data used in the experiments. At the same time, Ethereum transaction behavior analysis and Ethereum community structure analysis are proposed to better understand the network’s characteristics, such as in-degree and out-degree relationships. Also, Ethereum Link Prediction Analysis is introduced to perform the graph construction and representation for the link prediction. The experimental results show some useful statistical characteristics of the Ethereum network in terms of the distribution of active addresses, traffic of Ether history per address, and the degree distribution. Also, we could achieve high accuracy from 80–90% on the link prediction task given the time-series snapshot graph as inputs.

The main contributions of this manuscript are as follows:

• We propose DANET: A **D**etailed **A**nalysis of Ethereum **Net**work on Transaction Behavior, Community Structure and Link Prediction framework as a unified framework to return various aspects of analysis to support the understanding of the Ethereum network.
• We study the matter of Ethereum transaction tracking from a network perspective (*i.e.,* the influential addresses and community structure) which gives a deeper understanding of Ethereum transaction records and could contribute to the long-term evolution of the blockchain.
• We model Ethereum transactional data in the form of a heterogenous attributed network that preserves all the transactions’ essential information with graph auto-encoders for Ethereum link prediction.
• We make the code and dataset available for research purposes at github.com/Anwar-Said/Link-Predictability-using-VGAE.

The rest of the paper is organized as follows. ‘Introduction’ outlines the Ethereum data analysis and network-based representation approaches. ‘Related Work’ discusses background and relevant literature. ‘The Proposed Framework: DANET’ presents the methodology used in this research. ‘Experiment Results’ provides the experimental results and relevant discussions. Finally, ‘Conclusions’ concludes the paper.

## Related Work

This section presents an overview of the recent advancements in Ethereum, Bitcoin, and Network representation, mainly divided into Ethereum data analysis and network representation. The first category of approaches involves studying Ethereum and Bitcoin data using different techniques, while the latter deals with learning network structures using deep learning (DL) based graph representation approaches.

### Ethereum data analysis

Recently, many methods have been proposed to explore the Bitcoin network. Gencer et al. (2018) analyzed the number of Bitcoin users having large balances and studied graph-based Union-Find algorithm for finding addresses matching best to individuals. The authors also studied whether Bitcoin is primarily used for saving or routine transactions. Karame, Androulaki & Capkun (2012) presented a scenario for spending and avoiding double payments in Bitcoin transactions, by calculating the average “standard deviation” time, “transaction acceptance” time and “block generation” time of the network.

Similarly, Chan & Olmsted (2017) used a transaction-based graph that was configured on each node to analyze the behavior of each address. They also clustered the nodes using the similarity of the graph. The study concluded that Ethereum’s new transaction input is independent of the output of past unspent transactions, unlike Bitcoin. Gencer et al. (2018) analyzed the distribution statistics of various blockchains by mining power distribution. The results have shown that 61% of the weekly mining power was shared by only three IDs, with 90% of the power being shared by 11 entities. Mining nodes’ integrity was also evaluated by calculating the block numbers in the node that resulted in blocks of ankles(blocks that most miners rejected). Koshy, Koshy & McDaniel (2014) found that Bitcoin clients are designed for data collection where clients actively connect with their peers and collect all broadcast data along with IP information. The authors analyzed Bitcoin traffic, looked for anomalous relay patterns, and mapped Bitcoin addresses to IPs using the collected data. Moreover, anonymity links in the Bitcoin network were discovered using the aggregation method proposed by Reid & Harrigan (2011). The aggregation method associates different bitcoin addresses with users by specifying multiple inputs, multiple outputs, regular transactions, and geographically co-located IP addresses within a period. By splitting the shared Bitcoin wallet into different units, Meiklejohn et al. (2013) worked on the identification of identities in the executed transaction chunk by introducing intelligent clustering. By using heuristics of participating payments and address changes, authors who identified approximately 3.4 million clusters were able to put nearly 2,000 names from them. Additionally, Ober, Katzenbeisser & Hamacher (2013) suggested a structural analysis technique for the prediction of graph anonymity of the Bitcoin transactions. The author used a global passive adversary that defines entities according to the linkability of a transaction. Global enemies were also using participatory payment and address to change reasoning.

After Bitcoin, Ethereum is perhaps the second most popular cryptocurrency-based network; both employ blockchain, a distributed ledger technology. Both Bitcoin and Ethereum are digital currencies; however, the fundamental aim of Ether (Ethereum transactional token) is to facilitate and monetize the operation of the smart contract and decentralized application platform, rather than establish itself as an alternative monetary system. While Bitcoin networks have been extensively investigated and analyzed in the previous literature, the recent emergence of Ethereum in 2015 has merely drawn attention from limited research, making it scarcely explored (Li et al., 2020). Some of the recent studies that are relevant to the Ethereum data analysis is discussed here.

Maeng, Essaid & Ju (2020) proposed a node discovery algorithm for the Ethereum network utilizing the P2P links discovery. Furthermore, they analyzed the collected Ethereum data to identify the relationship between nodes, heavily connected nodes, and nodes geo-distribution. Farrugia, Ellul & Azzopardi (2020) proposed an XGBoost based classification algorithm for detecting the illicit accounts on the Ethereum network. Their dataset comprised 2,179 illicit accounts flagged by the Ethereum community and 2,502 normal accounts. They have identified that top features associated with illicit activities include ‘Time diff between first and last(Mins)’, ‘Total Ether balance’, and ‘Min value received’. Li et al. (2020) highlighted that all cryptocurrency and crypto-token transactions are permanently recorded on distributed ledgers and are publicly accessible, allowing for the development of a transaction graph and the analysis of connections between transaction graph characteristics and crypto price dynamics. They used the principles of persistent homology and functional data depth to study Ethereum crypto-tokens, particularly investigating price anomaly predictions and hidden co-movement between tokens. Using topological data analysis and functional data depth into blockchain data analytics, they discovered that the Ethereum network could provide valuable insights on price changes of crypto-tokens that are otherwise largely inaccessible with conventional data sources and traditional analytic methods. Xie et al. (2021) proposed to model Ethereum transaction records with a time-series snapshot network (TSSN) that captures the transactions’ spatial and temporal aspects. The network was traversed using the temporal biased walk (TBW) algorithm that effectively embeds accounts *via* their transaction records. They further explored two problems: phishing node classification and link prediction using a number of graph embedding algorithms. This study, however, lacks the analysis of the global Ethereum transaction network. Closest to our research would be the study by Wu et al. (2021) where the community detection problem was examined in both the Bitcoin and Ethereum networks. The low-rank community detection algorithm proposed by Wai et al. (2018) was used to detect communities in the Ethereum network. However, their study represented the Ethereum network as a graph of EoAs (users) and CAs (contracts) nodes since their objective was to identify sub-communities. Our research, on the other hand, also considers the Ethereum transactions as well.

### Network representation and link prediction

Learning network structure has received considerable attention in the last few years due to its wide range of applications, including recommender systems, molecular structures, biological systems, and various physical systems (Cai, Zheng & Chang, 2018). Since the network structure is unordered, classical machine learning and DL approaches are not directly applicable. The DL application on graphs was first presented by Scarselli et al. (2009) where Graph Neural Networks (GNNs) was proposed. This idea was later refined and extended by Gallicchio & Micheli (2010) and Kipf & Welling (2016a). GNNs methods generally involve several DL de facto standards such as random walks over networks, convolutions, recurrent neural networks, adversarial networks, message passing and autoencoders (Cai, Zheng & Chang, 2018; Hamilton, Ying & Leskovec, 2017; Zhang, Cui & Zhu, 2020; Said et al., 2020). These methods work in several settings in both supervised and unsupervised fashions. Various tasks can be performed over networks using these approaches, such as graph classification, node classification and link prediction (Bojchevski & Günnemann, 2018; Kipf & Welling, 2016b; Ahmed, Hassan & Shabbir, 2020). In the Ethereum network perspective, link predictability defines the ability to identify future transactions between two addresses. In other words, link prediction is a problem of identifying potential or missing links in a network.

From a network perspective, the link prediction task is a widely studied problem where its approaches consist of three categories: heuristics methods, graph embedding methods, and feature learning methods. The heuristics methods usually compute node similarities using graph-theoretic methods and use them as a likelihood of links (Zhang et al., 2020). Among which preferential attachment (Barabási & Albert, 1999), Jaccard coefficient (Liben-Nowell & Kleinberg, 2007), and Katz index (Katz, 1953) are well-known methods. Graph embedding methods involve learning free-parameter node embeddings based on the predefined network in a transductive setting where they cannot be generalized on unseen nodes (Grover & Leskovec, 2016; Hamilton, 2020). The third category involves the powerful and recently emerged Graph Neural Networks (GNNs) methods which learn node features using message passing mechanism and generalize well on unseen nodes (Kipf & Welling, 2016a; Kipf & Welling, 2016b; Said et al., 2021; Bojchevski & Günnemann, 2018; Hamilton, Ying & Leskovec, 2017). In a supervised setting, Graph Auto Encoders (VGAE) is largely adopted specifically for link prediction (Kipf & Welling, 2016b). In link prediction, VGAE learns node embeddings in an unsupervised fashion with a negative sampling approach (Yu et al., 2018). Kipf & Welling (2016b) introduced an unsupervised framework for learning graph-structured data with variational auto-encoders and latent variables. These methods have shown promising results and are now considered to be powerful tools for learning the graph-structured data (Zhang, Cui & Zhu, 2020; Said et al., 2020).

Unlike the existing works, we propose the framework named DANET to provide the Detailed Analysis of Ethereum Network on Transaction Behavior, Community Structure, and Link Prediction framework as a unified platform. Particularly, we adopt a unique approach to represent Ethereum data in the network form in the graph structure, allowing us to observe several exciting properties of the Ethereum network. We also considered the link predictability task on the constructed network and deployed VGAE (Kipf & Welling, 2016b), a powerful GNNs based learning model that yields outstanding link prediction results. We show that the Ethereum network consists of an exciting community structure, following the phenomenon of real-world networks.

##  The Proposed Framework: DANET

**Figure 2:**
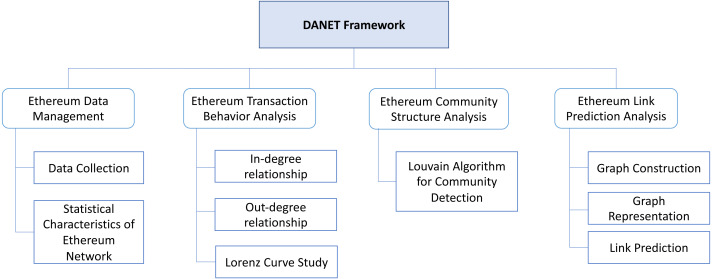
The proposed DANET framework architecture.

As shown in Fig. 2, to comprehensively analyze the Ethereum network and transaction records, we propose a consolidated framework: DANET, which includes four main modules to deliver the different analysis results. (1) Ethereum Data Management: to collect Ethereum transactional data for the experiments and compute the statistical characteristics of the Ethereum network; (2) Ethereum Transaction Behavior Analysis: to investigate the transaction behavior such as in- and out-degree relationships; (3) Ethereum Community Structure Analysis: to identify the trait of Ethereum community structure; (4) Ethereum Link Prediction Analysis: to evaluate the effectiveness of our framework on the Ethereum link prediction task. The details of each component are elaborated in the following subsections.

### Ethereum data collection

For data collection, we synced the Ethereum full node to collect all the historical transactional data. We used a spark cluster with one master node and two worker nodes with Ubuntu 16.04 having 40 GB RAM on each machine and an Internet connection of 10Mbps. We used geth (https://geth.ethereum.org/). Ethereum client as a full node to collect all the historical blocks data. This node took 11 days to collect data till 2018. We used the web3 API to send RPC requests to the Ethereum node. Web3 is an Ethereum compatible JavaScript API that implements the general JSON RPC specification. JSON-RPC is a transport-agnostic protocol that can be used over sockets and HTTP. We defined the RPC port and address while configuring the Ethereum node. We used *web*3.*eth*.*getBlock*(*id*, *true*) to retrieve blocks and extract transaction information from each block, and save the extracted information to a PostgreSQL database. The total collected Ethereum transactions data was from “2015-08-07″to “2019-01-01″comprising 189 million transactions in 55 blocks.

### Ethereum transaction behavior analysis

We used the Ethereum transactions dataset used by Muzammal et al. (2019). The dataset is 200 MB in size and was first downloaded and processed for understanding the Ethereum network. The raw data can be downloaded from Google BigQuery (https://tinyurl.com/7bmh3xkf). We constructed a directed Ethereum Transaction Featured Network (ETFN) where vertices represent addresses and edges represent the relationship in terms of transaction among the vertices. We also use the number of transactions among the pair of addresses (nonce), and the transferred amount (value) as a feature set over each edge to preserve transaction information. Formally, our ETFN is a attributed directed graph $\mathcal{G}=(\mathcal{V },\mathcal{E})$ where $\mathcal{V }=&lcub; {v}_{1},{v}_{2},{v}_{3},&hellip; ,{v}_{n}&rcub; $ and $\mathcal{E}=&lcub; {e}_{1},{e}_{2},{e}_{3},&hellip; ,{e}_{m}&rcub; $ where *n* = |*V*|, *m* = |*E*|. Also, we define *e* = (*u*, *v*, *w*) where *u* and *v* represent two nodes in $\mathcal{V }$, and *w* represents the weight of the edge between these two nodes .

### Ethereum community structure analysis

Exploring the community structure of a network plays a vital role in understanding the underlying network structure. There is no universal definition of a community within a network. However, it is widely accepted that the community represents a sub-group of vertices that are densely intra-connected and sparsely interconnected with the rest of the network (Said et al., 2018). A community represents a set of individuals with common interests within a network. For example, in a protein-protein interaction network, proteins having common functionality may belong to the same community. A community may represent a particular region of the brain having dense neurons connectivity in a brain network. Similarly, in a transaction network, a community represents individuals who frequently make transactions with each other. Exploring a transaction network’s communities can reveal individuals’ potential and valuable information regarding their transaction patterns and time slots (if the network is time-variant) (Newman & Girvan, 2004; Newman, 2006; Said et al., 2019).

Due to numerous applications in a wide range of real-world settings, community detection has caught the research community’s special attention, especially the Louvain community detection algorithm (Blondel et al., 2008). The Louvain algorithm is a greedy method based on the optimization of the modularity measure that has been extensively used to identify communities in crypto-currency networks, such as that of Bitcoin (Remy, Rym & Matthieu, 2017; Zhang, Wang & Zhao, 2020; Gavin & Crane, 2021). While the Bitcoin network has some differences from the Ethereum network, it makes sense to follow similar protocols widely used to analyze these cryptocurrency networks. The Louvain algorithm is a greedy method based on optimization of the modularity measure, which can be defined for a simple undirected network as follows.

(1) \begin{eqnarray*}\mathcal{Q}=\sum _{c=1}^{k} \left[ \frac{{\mathcal{E}}_{c}}{\mathcal{E}} -{ \left( \frac{{\deg \nolimits }_{c}}{2\mathcal{E}} \right) }^{2} \right] \end{eqnarray*}

In the above equation, k is the total number of communities, ${\mathcal{E}}_{c}$ is the total number of edges, deg_*c*_ indicates the total degree in the community *c*, and $\mathcal{E}$ is the total edges in the network. The modularity value ranges between [-1,1], where the highest value indicates a good community structure and vice versa. The negative value means no community structure in the network. The value approaches zero if all the vertices are assigned to a single community (Newman, 2006).

The Louvain algorithm optimizes the modularity value of the network and consists of two phases. The first phase assigns a different community to each node and then attractively combines each node to its neighbors’ community and evaluates the modularity score. In case of improvements in the modularity score, nodes are merged into a single community. This process is repeated until there is no gain in the modularity score. In the second phase, the first phase communities are compressed to a single node where the internal edges are used as self-links and repeat the first step. Once no further improvements are found, the algorithm stops and returns the identified community structure. Louvain community detection algorithm is known to be one of the scalable algorithms having *O*(*n*log*n*) where *n* is the number of nodes (Blondel et al., 2008).

### Ethereum link prediction analysis

#### Graph construction

To perform link predictability on our Ethereum Transaction Featured Network (EFTN), we employ the Variational Graph Auto-encoder (VGAE) (Kipf & Welling, 2016b) as our primary model. Given a graph $\mathcal{G}=(\mathcal{V },\mathcal{E})$, with $N={|}\mathcal{V }{|}$ vertices, let *A* ∈ {0, 1}^*N*×*N*^ denote the adjacency matrix of $\mathcal{G}$ where *A*_*ij*_ = 1 if *v*_*i*_ and *v*_*j*_ are neighbors and 0 otherwise. Let **D**^*N*×*N*^ denote the degree matrix of $\mathcal{G}.\mathbf{D}$ is a diagonal matrix where its diagonal values *D*_*i*,*i*_ equals the degree of *v*_*i*_. Similarly, let $A{\mathbf{D}}^{- \frac{1}{2} }\mathbf{A}{\mathbf{D}}^{- \frac{1}{2} }$ be the normalized adjacency matrix. Let *N*_*i*_ denote the network neighborhood of a vertex *v*_*i*_, *X*^*N*×*d*^ represents node features matrix and *z*_*i*_ is a stochastic latent variable summarized in an *N* × *d* matrix *Z*. Note that *N*_*i*_ can be either complete *v*_*i*_’s neighborhood or it can be generated through a neighborhood sampling strategy $\mathcal{S}$, where the sampling strategy is a technique to randomly select a subset of vertices or edges from the original graph. The network embedding is a function $\mathrm{&phi;}:V&rarr; {\mathcal{R}}^{d}$ that maps the vertices to a feature representation. Here *d* indicates the dimension of our feature presentation for each vertex. Therefore, *ϕ* is a matrix of size *N* × *d* parameters.

#### Graph representation

Variational Graph Auto-Encoders (VGAE) is a GCN-based link prediction method over networks. The algorithm has recently been adopted to learn graph representation of the Bitcoin network (Shah et al., 2021; Zhang et al., 2021). VGAE’s framework first learns vertex embeddings of the entire network using GCNs, and then the aggregation of source and target nodes is performed to predict the target link (Kipf & Welling, 2016b). The method uses the standard notion of variational auto-encoders while learning µand *σ* to generate the desired output. The architecture includes two layers of GCNs where the first layer generates the latent variables **Z** and the second layer generates µand *σ*. Then the standard parameterization trick is used to calculate **Z**. Given the input **A** and **X**, the first layer of GCN is defined as follows.

(2) \begin{eqnarray*}X=GCN(\mathbf{X,A})=ReLu(A\mathbf{X}{\mathbf{W}}_{0}).\end{eqnarray*}

The second layer of GCN generates µand *σ* from Xˆ as follows.

(3) \begin{eqnarray*}\mu =GC{N}_{\mu }(\mathbf{X},\mathbf{A})=\boldsymbol{\^{A} }X{\mathbf{W}}_{1}\end{eqnarray*}

(4) \begin{eqnarray*}\sigma =GC{N}_{\sigma }(\mathbf{X},\mathbf{A})=\boldsymbol{\^{A} }X{\mathbf{W}}_{1}\end{eqnarray*}

where **W**_0_ and **W**_1_ are the model weight matrices. Each element **W**_*i*,*j*_ in **W**_0_ and **W**_1_ represents the weight of the edge between the *i*th vertex and the *j*th vertex.

The decoder model is simply *A*=*σ*(*zz*^⊺^), where *σ*(.) is a logistic sigmoid function. The overall encoder–decoder model is defined as follows.

(5) \begin{eqnarray*}q({z}_{i}{|}\mathbf{X},\mathbf{A})=N({z}_{i}{|}\mu ,\text{diag}(\sigma )^{2})\end{eqnarray*}

and the decoder is represented as

(6) \begin{eqnarray*}p({\mathbf{A}}_{ij}=1{|}{z}_{i},{z}_{j})=\sigma ({z}_{i}^{\top }{z}_{j}).\end{eqnarray*}

The loss function of VGAE is similar to the standard variational auto-encoders and is defined below.

(7) \begin{eqnarray*}\mathcal{L}={\mathbb{E}}_{q(\mathbf{Z}{|}\mathbf{X},\mathbf{A})} \left[ \log \nolimits p(\mathbf{A}{|}\mathbf{Z}) \right] -\text{KL} \left[ q(\mathbf{Z}{|}\mathbf{X},\mathbf{A}){|}{|}p(\mathbf{Z}) \right] .\end{eqnarray*}

The first part is the reconstruction loss between the original and the constructed adjacency matrix, while the second part is the KL divergence for *p*(**Z**) = *N*(0, 1).

#### Link prediction

This section describes the experimental setup and results for the link predictability task on our Ethereum network. Recall that our EFTN network consists of 2.7 million vertices and 4.6 million edges. Also, the network is attributed where it contains nonce and value as features on each edge. For nodes’ features, we used one-hot degree encoding; however, we fixed the size of the feature vector to 100.

**Figure 3:**
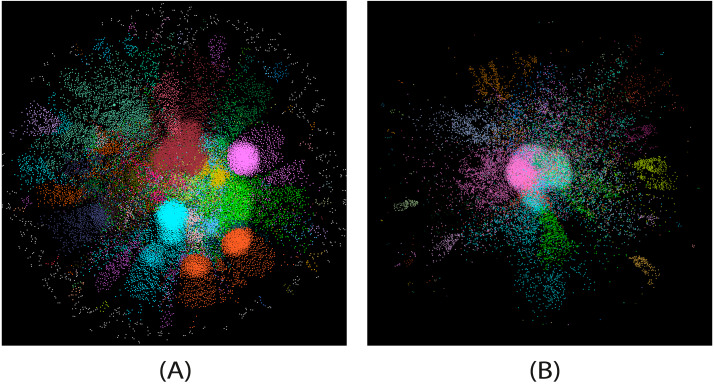
The visualizations of ${\mathcal{G}}_{1}$ (A) and ${\mathcal{G}}_{2}$ (B) EFTN networks.

We observed that few nodes (less than 10) had large degrees, playing the role of hubs in the network. Thus, to avoid sparsity in our feature matrix, we fixed the size and assigned a degree of 100 if a node’s degree is greater than 100. Due to the memory limitation, we constructed two different networks while choosing a chunk from the whole data. We only considered 20 days of transactions: from 2016-12-1 to 2016:12:20 where the total number of records was 0.42 million. The first 15 days comprise around 0.210 million transactions, while the remaining five days have 0.211 million transactions. We constructed two networks ${\mathcal{G}}_{1}$ and ${\mathcal{G}}_{2}$ separately from this data. The numbers of nodes and edges in ${\mathcal{G}}_{1}$ were 33,989 and 53,261, respectively, while there were 37,175 nodes and 56,987 edges in ${\mathcal{G}}_{2}$. Please note that we consider the chunk from the data randomly; however, we believe that the slice of data at any point can be considered and would produce similar results. Also, we consider both the networks as undirected, as we want to predict a transaction among two addresses made from either side. We show the visualizations of both the constructed networks in Fig. 3.

We considered a two-layer network in GCN architecture and considered 100 and 8 neurons in the encoder layer. As mentioned previously, our decoder layer is simply the dot product of the learned feature vectors of the corresponding vertices. We used negative sampling for preparing the training and test data (Mikolov et al., 2013). The ratio of the train and test splits was set to 67:33 accordingly. We set the number of epochs to 100, and the learning rate to 0.001.

## Experiment Results

In this section, we provide a complete set of analyses based on the Ethereum network as follows.

### Statistical characteristics of ethereum network

As shown in Table 2A, we can notice that the majority of addresses (88%) are associated with less than 10 transactions each. Also, 39 addresses are frequently used on the network and are associated with at least 50, 000 transactions. On the other hand, 32% of addresses participate in a transaction only once. There are also six active addresses participating in over 1, 000, 000 (30%) transactions. We investigate the six active addresses: ENS-Registrar, YoCoin, Bittrex_2, Acronis_Contract, Poloniex_1, and Kraken_5 and found them to be contract addresses.

**Table 2:** (A): The distribution of active addresses. Min and max represent the minimum and maximum of the transactions. (B): The breakdown of per address transactions.

min	max	#addresses
1	2	1,115,238
2	4	1,509,244
4	10	1,102,949
10	100	364,406
100	1,000	47,711
1,000	5,000	3,307
5000	10,000	219
10,000	50,000	236
50,000	100,000	39
100,000	500,000	40
500,000	1,000,000	8
1,000,000		6

min	max	#addresses
1	2	1,700,413
2	4	1,066,002
4	10	320,416
10	100	194,338
100	1,000	33,090
1,000	5,000	1,546
5000	10,000	114
10,000	50,000	127
50,000	100,000	19
100,000	500,000	21
500,000	1,000,000	3
1,000,000		2

Similarly, as shown in Table 2B, 1, 700, 413 (49%) support transactions were received only once in history, concluding that most wanted to remain anonymous as they changed their addresses after each transaction. Considering the distribution of total transferred transactions per address (Table 3), we noticed that less than 10 transactions were received from 156, 304 (90%) addresses. The study found that the total number of Ethers received from most addresses was barely significant.

**Table 3:** Breakdown of total transactions sent per address.

min	max	#addresses
1	2	1,319,452
2	4	984,028
4	10	419,211
10	100	156,304
100	1,000	16,630
1,000	5,000	1,069
5000	10,000	91
10,000	50,000	112
50,000	100,000	20
100,000	500,000	20
500,000	1,000,000	5
1,000,000		2

Table 4 shows that 28% of addresses send less than one accumulated Ether in a transaction. In its history, 48% of addresses send less than 10 Ether, and 63% of addresses receive less than 100 Ether.

**Table 4:** Breakdown of outgoing accumulative Ether history per address.

Total Ether (≥)	Total Ether (<)	Number of addresses
0	1	917,327
1	10	695,867
10	100	469,766
100	1,000	224,543
1,000	10,000	548,540
10,000	50,000	39,202
50,000	100,000	899
100,000	500,000	648
500,000	5,000,000	128
5,000,000	50,000,000	25
50,000,000		1

Table 5 shows that 1 or less Ether was received by 32% of all addresses (1, 088, 717), less than 10 Ether were received by 58% of addresses, and less than 100 Ether received by 75% of addresses.

**Table 5:** Breakdown of incoming accumulative Ether history per address.

Total Ether (≥)	Total Ether (<)	Number of addresses
0	1	1,088,717
1	10	863,216
10	100	537,756
100	1,000	242,315
1,000	10,000	546,260
10,000	50,000	36,344
50,000	100,000	717
100,000	500,000	607
500,000	5,000,000	131
5,000,000	50,000,000	26
50,000,000		2

Table 6 shows that nearly 96% of the addresses’ current (May 15, 2017) balance is less than 10 Ether, but this number drops to 82% when looking at the maximum balance that can be seen during the life of these addresses. Table 6 states that only 1, 049 (0.2%) addresses have a balance of 10, 000 or more.

**Table 6:** The breakdown of Ether balance per address (until May 15, 2017).

Total Ether(≥)	Total Ether (<)	Number of addresses
0	0.01	2,493,480
0.01	0.1	288,026
0.1	1	193,895
1	10	193,057
10	100	87,533
100	1000	28,418
1000	10,000	6,079
10,000	50,000	781
50,000	100,000	98
100,000	500,000	119
500,000	2,500,000	35
2,500,000		16

Table 7 represents the distribution of the transaction sizes of the network. At other times, many transactions are very small, and it is noticeable that less than 1 Ether has been received by 53% of transactions. Similarly, considering medium-sized quantities, less than 10 Ether were received by 88% of transactions. Moreover, Table 7 shows that only 1, 788 transactions received greater than 50, 000 Ether.

**Table 7:** Ethereum network’s transaction size distribution.

Total Ether(≥)	Total Ether(<)	Number of addresses
0	0.001	6,552,962
0.001	0.1	4,360,858
0.1	1	8,585,043
1	10	12,544,316
10	100	2,358,529
100	1,000	1,245,886
1,000	10,000	607,476
10,000	50,000	10,815
50,000	100,000	1,040
100,000	500,000	696
500,000	2,500,000	41
2,500,000		2

### Ethereum transaction behavior analysis

**Figure 4:**
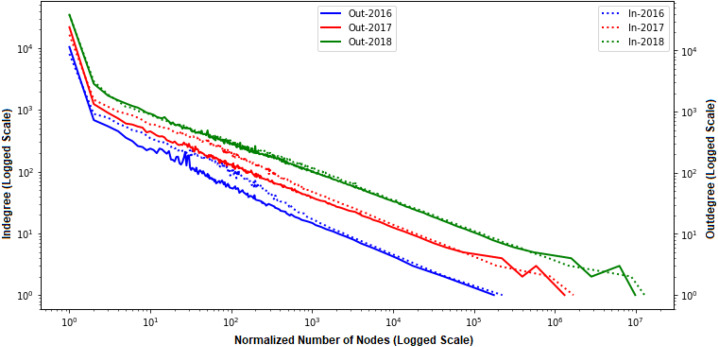
Degree distributions of various time periods.

We analyzed the transaction flow by breaking the data into two phases, in-degree and out-degree relationships. We considered each year (2016, 2017, and 2018) as a single phase and constructed the corresponding network. Since the network grows over time, we are also interested in measuring network growth. We first measured our constructed Ethereum networks’ degree distributions, as shown in Fig. 4. from the distribution, we approximated to the power law and observed that both the out-degree and in-degree are relatively uniform. Also, the number of nodes and their degrees are increasing with time passing.

**Figure 5:**
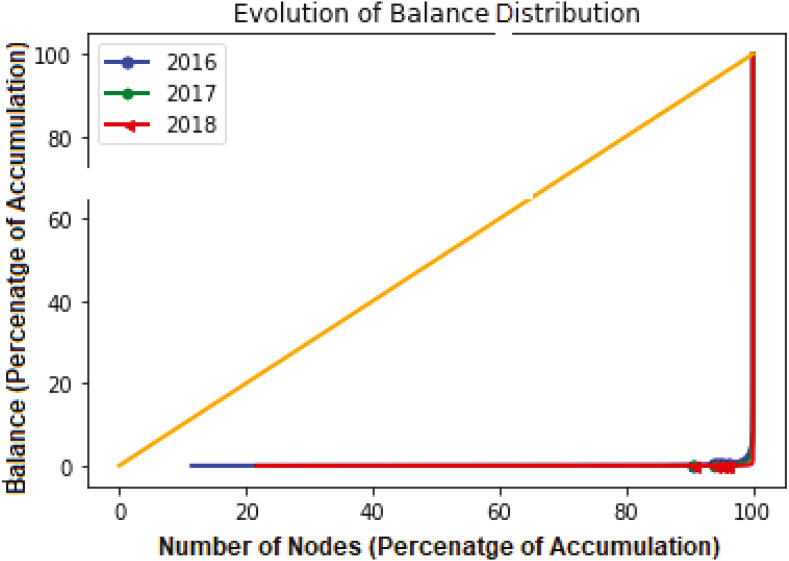
The Lorenz curve of the address balance at other moments.

Figure 5 draws a Lorenz curve (a graphical representation of the Gini Coefficient) to additionally characterize the evolution of the order distribution and calculate the “Gini Coefficient” with other timestamps. In order to measure the inequalities that present in the breakdown of wealth, we used such a scale because it is also used to calculate the heterogeneity of the empirical data. In general, the Gini coefficient is calculated as follows:

\begin{eqnarray*}{G}_{c}= \frac{2\sum _{j=1}^{t}j{x}_{j}}{t\sum _{j=1}^{t}{x}_{j}} - \frac{t+1}{t} . \end{eqnarray*}

Here, *x*_*j*_ is the *j*th sample from *t* data points, and *x*_*j*_ is ordered monotonically, *i.e., x*_*j*_ ≤ *x*_*j*+1_.*G*_*c*_ = 1, implies complete inequality and *G*_*c*_ = 0 indicates perfect equality in wealth distribution, *i.e.,* all nodes have the same wealth amount.

**Figure 6:**
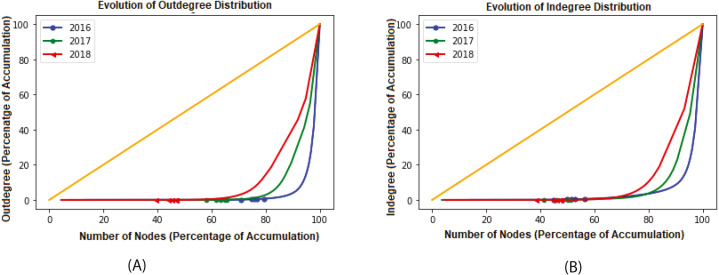
Different time frames of Lorenz curves for out-degree and in-degree.

Figure 6A shows that the Ethereum network is changing with time. The line of equality is indicated using the Yellow line. If other lines get closer to it, this means that the system is moving to equality. As we see from the figure, EFTN moves towards equality as the curves get closer to the Lorenz curve as time passes. The Gini coefficient computed for out-degree each year was *G*^*out*^ ≃ 0.96, *G*^*out*^ ≃ 0.92 and *G*^*out*^ ≃ 0.85 respectively for years 2016, 2017 and 2018.

Similar behavior for network in-degree was also observed, as shown in Fig. 6B. Gini Coefficient values were *G*^*in*^ ≃ 0.95, *G*^*in*^ ≃ 0.90 and *G*^*in*^ ≃ 0.83 for each year 2016, 2017 and 2018 respectively. For both in and out degrees, the Gini Coefficient values are close to 1 for each year under consideration. This implies large inequality among sending and receiving transactions distributions. Apart from the in-degree and out-degree distributions, we can observe lacking balance among addresses, as shown in Fig. 5. The figure indicates only a few addresses own a major part of the Ethers representing perfect inequality in the distribution.

**Figure 7:**
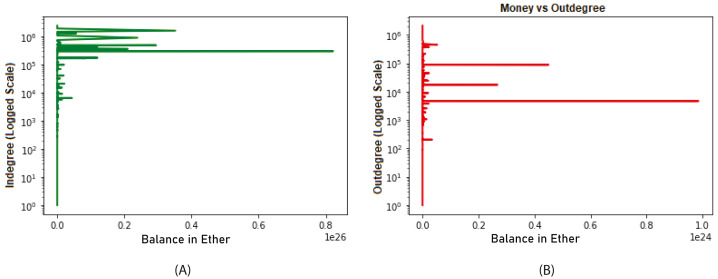
Relation between balance and in- and out-degrees (until 2018-04-25).

We analyzed nodes with a high degree compared to other nodes in the network. Nodes with higher order are assumed to have higher balances. In Fig. 7A, it is noticed that higher proportion is associated with higher in-degree nodes till date 2018-04-25. However, there is no relation between out-degree and the balance as depicted in Fig. 7B. Therefore, we concluded that the distribution of the Ether is associated more with the in-degrees rather than the out-degrees.

### Ethereum community structure analysis

**Figure 8:**
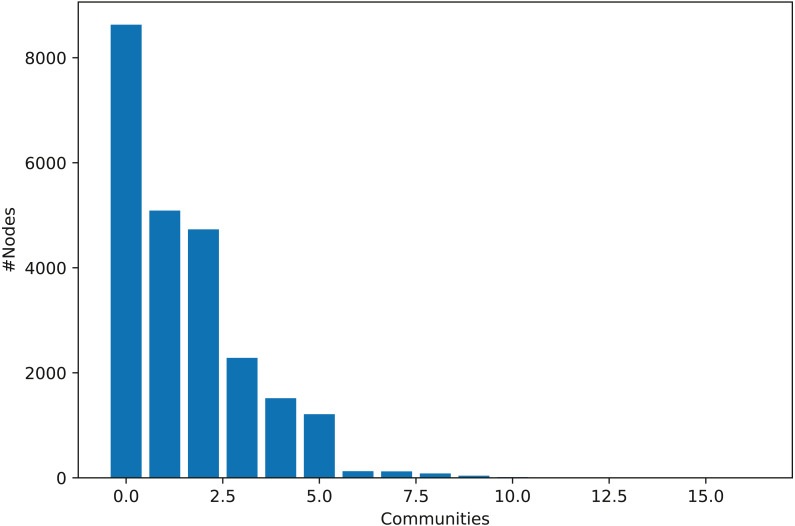
Histogram representation of EFTN community structure.

To explore our ETFN network’s community structure, we deployed the Louvain algorithm in our experimental setting. The histogram representation of the network community structure is shown in Fig. 8 depicting an exciting observation. On the *x*-axis, the number of communities is shown, while the *y*-axis represents individuals’ count (addresses in this case) in each community. We can see that the entire network comprises five major communities while a few other smaller communities. The community distribution shows quite interesting observation resembling the community distribution of most of the real-world networks (Said et al., 2018). Moreover, one central community contains many influential addresses and covers most of the network (around 30%). These results indicate that EFTN consists of some excellent community structure, and thus, various network theory measures can be deployed to mine further hidden information from it.

### Ethereum link prediction analysis

**Figure 9:**
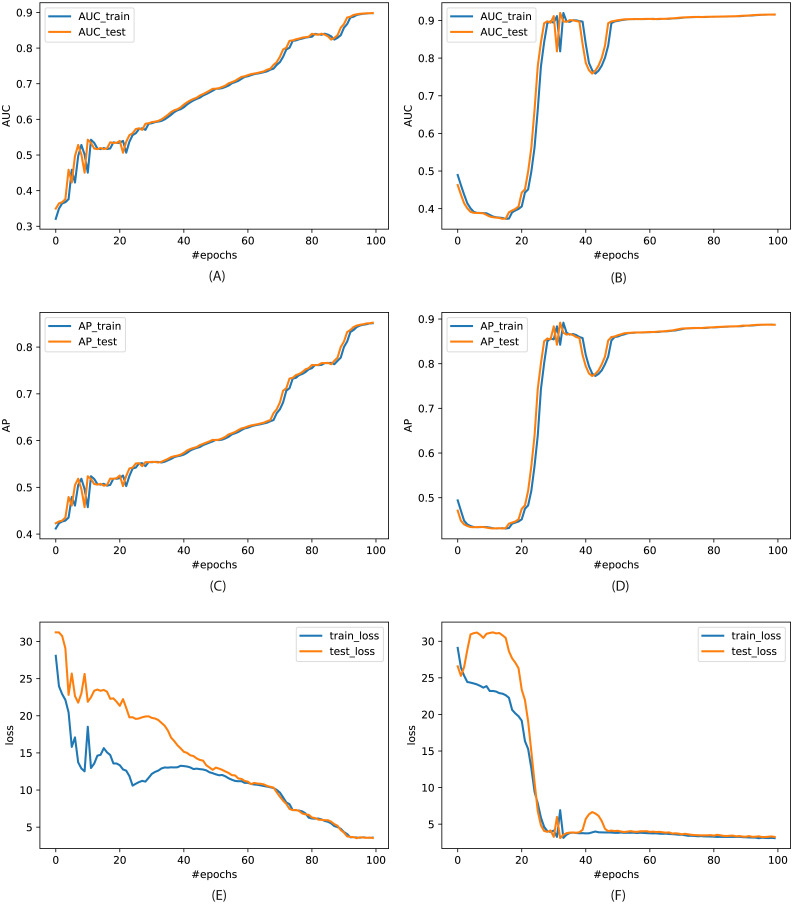
(A & B): Area Under the Curve (AUC) of VGAE model on both ${\mathcal{G}}_{1}$ and ${\mathcal{G}}_{2}$ for 100 epochs. (C & D): The performance in terms of Average Precision (AP). (E & F): The corresponding loss curves.

This section considers standard Area Under the Curve (AUC) and Average Precision (AP) matrices for evaluation. The performance of VGAE in terms of AUC and AP on both the networks is shown in Fig. 9. We can see that the VGAE model has shown outstanding performance while achieving 87.6% AUC on ${\mathcal{G}}_{1}$ and 91.59% AUC on ${\mathcal{G}}_{2}$ networks. Similarly, it shows 88.28% and 88.5% AP on ${\mathcal{G}}_{1}$. These results demonstrate the effectiveness of VGAE on the Ethereum transaction data. Furthermore, we observe that both the networks have similar statistics and structures; ergo, the performance of the models is also quite closed using both evaluation metrics.

## Discussion and Future Work

In this study, we provided a set of analyses based on the Ethereum network as follows. First, we noticed that most Ethereum addresses are associated with a few transactions when analyzing the outgoing and incoming accumulative Ether history per address. Second, we observed that the number of nodes and their degrees during 2016-2018 increased with time regarding the measurement of the in-degree and out-degree transaction relationship. Specifically, we discovered that the distribution of Ether is more associated with the in-degrees rather than out-degrees. Third, we recognize five major communities from the entire network. Lastly, the performance of VGAE on Ethereum’s link prediction in terms of area under the curve and average precision matrices is outstanding, with over 80% on sub-networks over time.

In the future, we plan to use our findings in this study as a groundwork for comparing the statistical features from more Ethereum data, examining the evolution of temporal properties in the transaction network, and gaining a better understanding of the complex interaction between the transaction network and the social network. In addition, we could investigate graph algorithms that can handle the community detection and link prediction problems altogether, using either traditional graph analysis (Lü & Zhou, 2011) or graph representation learning methods (Choong, Liu & Murata, 2018; Liu et al., 2020). Also, this study could lay the direction for further research on optimizing and managing the optimal usage of the Ethereum network for better network maintenance. Finally, more recent data could be collected and processed to investigate the evolution of the network behavior over time.

## Conclusions

In this paper, we proposed a Detailed Analysis of Ethereum Network on Transaction Behavior, Community Structure and Link Prediction framework (DANET) to track the evolution of Ethereum transactional data from the perspective of graph analysis. Also, we investigated wealth distribution over Ethereum in terms of network degree and explored the network’s community structure showing a piece of exciting information. We further performed link prediction using variational graph auto-encoders on a small set of transaction data. The model showed impressive prediction accuracy on the link prediction task. By examining these graphs through several metrics, we gain many new observations and insights, which could assist the understanding of the Ethereum network.

## Additional Information and Declarations

### Data Availability

The following information was supplied regarding data availability: The code and dataset are available at GitHub: https://github.com/Anwar-Said/Link-Predictability-using-VGAE.
